# Understanding health behaviours in context: A systematic review and meta-analysis of ecological momentary assessment studies of five key health behaviours

**DOI:** 10.1080/17437199.2022.2112258

**Published:** 2022-09-15

**Authors:** Olga Perski, Jan Keller, Dimitra Kale, Bernard Yeboah-Asiamah Asare, Verena Schneider, Daniel Powell, Felix Naughton, Gill ten Hoor, Peter Verboon, Dominika Kwasnicka

**Affiliations:** a Department of Behavioural Science and Health, University College London, London, United Kingdom; b Department of Education and Psychology, Freie Universität Berlin, Berlin, Germany; c Curtin School of Population Health, Curtin University, Perth, Australia; d Health Psychology, Institute of Applied Health Sciences, University of Aberdeen, Aberdeen, United Kingdom; e Rowett Institute, University of Aberdeen, Aberdeen, United Kingdom; f Behavioural and Implementation Science Research Group, School of Health Sciences, University of East Anglia, Norwich, United Kingdom; g Department of Work and Social Psychology, Faculty of Psychology and Neurosciences, Maastricht University, Maastricht, The Netherlands; h Faculty of Psychology, Open University, Heerlen, The Netherlands; i Faculty of Psychology, SWPS University of Social Sciences and Humanities, Wroclaw, Poland; j NHMRC CRE in Digital Technology to Transform Chronic Disease Outcomes, Melbourne School of Population and Global Health, University of Melbourne, Melbourne, Australia

**Keywords:** Ambulatory assessment, ecological momentary assessment, experience sampling, health psychology, systematic review, meta-analysis

## Abstract

Ecological Momentary Assessment (EMA) involves repeated, real-time sampling of health behaviours in context. We present the state-of-knowledge in EMA research focused on five key health behaviours (physical activity and sedentary behaviour, dietary behaviour, alcohol consumption, tobacco smoking, sexual health), summarising theoretical (e.g., psychological and contextual predictors) and methodological aspects (e.g., study characteristics, EMA adherence). We searched Ovid MEDLINE, Embase, PsycINFO and Web of Science until February 2021. We included studies focused on any of the aforementioned health behaviours in adult, non-clinical populations that assessed ≥1 psychological/contextual predictor and reported a predictor-behaviour association. A narrative synthesis and random-effects meta-analyses of EMA adherence were conducted. We included 633 studies. The median study duration was 14 days. The most frequently assessed predictors were ‘negative feeling states’ (21%) and ‘motivation and goals’ (16.5%). The pooled percentage of EMA adherence was high at 81.4% (95% CI = 80.0%, 82.8%, *k *= 348) and did not differ by target behaviour but was somewhat higher in student (vs. general population) samples, when EMAs were delivered via mobile phones/smartphones (vs. handheld devices), and when event contingent (vs. fixed) sampling was used. This review showcases how the EMA method has been applied to improve understanding and prediction of health behaviours in context.

## Introduction

Andy Warhol wrote: ‘They always say time changes things, but you actually have to change them yourself.’ This holds true for changing key health behaviours: increasing exercise and reducing time spent sitting, eating healthily, drinking less alcohol, stopping smoking, and having safe sex. In the health psychology domain, researchers have traditionally relied on one-off assessments of psychological constructs (e.g., motivation, self-efficacy), contextual factors (e.g., weather), and health behaviours. Researchers typically ask participants if they want to change their health behaviour(s), if they feel confident to do so, and if they perceive any specific change barriers or facilitators. We also tend to ask participants to retrospectively recall the average frequency of their health behaviour(s) over longer time periods (e.g., ‘On average, how many times per week do you exercise?’). In the early 1980s, a method referred to as *experience sampling* (or Ecological Momentary Assessment; EMA) was introduced (Larson et al., 1980), which involves repeated (often technology-mediated), real-time measurements of cognitions, emotions, environmental contexts, and behaviours in people’s daily lives (Stone & Shiffman, 1994).

This new method has revolutionised health psychological research: through relying on real-time (as opposed to retrospective) assessments of variables of interest, findings from EMA studies have provided a more precise and reliable understanding of how health behaviours unfold over time and in context, also mitigating methodological issues such as recall bias (Reichert et al., 2020). For example, participants are better at recalling emotions and cognitions at hourly or daily compared with weekly or monthly retrospective reports (Shiffman et al., 2007). In the four decades since its inception, the EMA method has been applied across many health behaviours. However, no review has synthesised findings from the many available EMA studies, summarising key theoretical and methodological aspects. Although prior systematic reviews have summarised methodological aspects of EMA studies (Cain et al., 2009; Colombo et al., 2019; de Vries et al., 2021; Degroote et al., 2020; Heron et al., 2017; Jones et al., 2019; Schembre et al., 2018; Wen et al., 2017), to the best of our knowledge, no available review has summarised psychological and contextual predictors of health behaviours measured via EMAs or compared methodological aspects across EMA studies focused on five key public health behaviours, which are leading causes of morbidity and premature mortality globally (Murray et al., 2020), including: physical activity and sedentary behaviour, dietary behaviour, tobacco smoking, alcohol consumption, and sexual health behaviour. We aimed to fill this gap by synthesising findings from EMA studies conducted across these five key health behaviours of interest.

### Theoretical considerations in EMA studies: studying dynamic health behaviour change within persons

To predict and explain health behaviours and inform the development of effective behaviour change interventions, theories of health behaviours must apply to individuals (Johnston & Johnston, 2013). However, most studies that aim to test or build health psychology theory are designed in such a way that they can only explain why people are different from one another (i.e., they capture between-person differences). *Ergodic processes* are those that are identical for groups and individuals, with the mean and variance of the process (e.g., motivation to exercise) remaining consistent over time. Inferences made from group-level estimates of psychological processes can only be validly applied to understanding individuals if the process of interest is *ergodic*. However, evidence from EMA studies shows that the ergodicity assumption rarely holds for psychological processes (Fisher et al., 2018). Calls have therefore been made to focus research efforts on both group- and individual-level change processes (Chevance, Perski, et al., 2020; Fisher et al., 2018; Hekler et al., 2019). For example, EMA studies have been used to capture the co-occurrence of psychological and/or contextual variables and health behaviours (‘synchronicity’; e.g., positive affect while eating), antecedents and consequences of health behaviours (‘sequentiality’; e.g., the lagged effect of intentions on physical activity), critical fluctuations in psychological or contextual variables and health behaviours (‘stability’ or ‘instability’), with a focus on individual-level change processes (Dunton, 2017).

In addition, one cannot consider behaviour change without considering time. Few health psychology theories explicitly refer to time in their conceptualisation of change processes (Scholz, 2019), such as specifying the timeframe within which change in a psychological or contextual variable is expected to lead to change to the target behaviour, and with what magnitude. This only scratches the surface of the importance of time for the understanding and prediction of health behaviour change: both the psychological or contextual variable and the behaviour are likely to have their own group- and individual-level variances (i.e., ‘stability’ or ‘instability’ over time) and covariances that may or may not be systematically associated with time of day, week, month, or year.

To test any clearly articulated health behaviour change theory, study designs that can reliably capture the dynamics of psychological and behavioural processes at the within-person level are required, followed by the use of a statistical or computational approach that robustly operationalises the theoretical model (Collins, 2006). EMA studies are well-suited for capturing such dynamics as these allow researchers to flexibly schedule real-time assessments at different temporal frequencies (e.g., daily, hourly). Study designs and prompting schedules vary across EMA studies, with the latter being triggered by time (e.g., fixed, random, quasi-random or stratified prompts) or event occurrence (e.g., after having smoked a cigarette). Due to recent technological advances, the dynamics of health behaviours can also be captured using passive and continuous sensing with portable and/or wearable devices, which can be used to trigger event-based assessments when some predefined threshold is reached (Ebner-Priemer et al., 2013; Giurgiu et al., 2020).

However, to the best of our knowledge, no available review has summarised what psychological and contextual predictors have been examined – and at what sampling frequency – in EMA studies of the five key health behaviours of interest.

### Methodological considerations in EMA studies: prompting schedules, incentives and adherence

Their theoretical benefits notwithstanding, EMA studies bring key methodological challenges for participants and researchers, including the burden associated with some prompting schedules (potentially leading to low adherence) (Reichert et al., 2020), a limited number of validated instruments for measuring state-like (i.e., dynamically fluctuating) psychological and contextual variables and health behaviours, and the requirement for researchers to master relatively sophisticated statistical modelling techniques, including multilevel/hierarchical regression models (Bolger & Laurenceau, 2013).

Several systematic reviews of EMA studies have been conducted across domains such as mental health (aan het Rot et al., 2012; Bell et al., 2017; Colombo et al., 2019; de Vries et al., 2021; Enkema et al., 2020; Gee et al., 2020; Goldschmidt et al., 2014; Haedt-Matt & Keel, 2011; Liu et al., 2019; Loo Gee et al., 2016; Mote & Fulford, 2020; Santangelo et al., 2014; Versluis et al., 2016; Walz et al., 2014; Yang et al., 2019), children/youth (Heron et al., 2017; Mason et al., 2020; Wen et al., 2017), older adults (Cain et al., 2009), individuals at risk of HIV exposure (Smiley et al., 2020), and specific health behaviours such as dietary behaviour (König, Emmenis, et al., 2021; Mason et al., 2020; Maugeri & Barchitta, 2019; Schembre et al., 2018), physical activity and/or sedentary behaviour (Degroote et al., 2020; Dunton, 2017; Papini et al., 2020; Romanzini et al., 2019) and substance use (Jones et al., 2019; Morgenstern et al., 2014; Soyster & Fisher, 2019; Votaw & Witkiewitz, 2021; Wray et al., 2014). In addition, several reviews have summarised rates of adherence to EMAs across studies focused on specific health behaviours (e.g., substance use) or populations (e.g., children and adolescents) – which have, on average, been estimated to fall between 71.6% to 79.0% of the total number of delivered EMAs (Cain et al., 2009; Colombo et al., 2019; de Vries et al., 2021; Degroote et al., 2020; Heron et al., 2017; Jones et al., 2019; Schembre et al., 2018; Wen et al., 2017). In addition, some of these behaviour- or population-specific reviews have looked at moderators of EMA adherence, showing, for example, that studies with subtance use dependent (vs. non-dependent) samples (Jones et al., 2019) and studies with higher (vs. lower) EMA sampling frequencies reported lower EMA adherence (Wen et al., 2017; Williams et al., 2021).

Although systematic reviews of EMA studies focusing on specific health behaviours are available, we lack a comprehensive summary of theoretical (e.g., psychological and contextual predictors) and methodological aspects (e.g., study designs, frequency of EMAs, incentives, EMA adherence) of EMA studies across key health behaviours, including physical activity and sedentary behaviour, dietary behaviour, tobacco smoking, alcohol consumption, and sexual health behaviour. The extent to which such theoretical and methodological aspects differ by target health behaviour remains an empirical question. Such information is useful for health psychology researchers planning the design of future EMA studies, identifying knowledge gaps, and providing a summary of best practice across research contexts, settings, and health behaviours. Although we acknowledge that our list of target health behaviours could beneficially be expanded to, for example, include medication adherence, healthcare seeking behaviour, and sleep, we were mindful when designing the review protocol that a large number of studies would likely be in scope and therefore opted to impose a boundary to only include key public health behaviours which are known to account for a considerable proportion of mortality and morbidity globally (Murray et al., 2020).

### The present study

The present systematic review and meta-analysis therefore aimed to showcase the current state-of-knowledge in EMA health behaviour research and identify knowledge gaps by summarising theoretical (e.g., psychological and contextual predictors) and methodological aspects (e.g., study settings, study designs, sample characteristics, study durations, frequency of EMAs, EMA prompting strategies, adherence to EMAs, incentive structures) of EMA studies across five key health behaviours.

## Methods

### Study design

This review adhered to the Preferred Reporting Items for Systematic Reviews and Meta-Analyses (PRISMA) checklist (Moher et al., 2009) and the American Psychological Association’s Meta-Analysis Reporting Standards (Cooper, 2020). A protocol was pre-registered on the Open Science Framework (www.osf.io/cmnvw) and on the international Prospective Register of Systematic Reviews (www.crd.york.ac.uk/prospero/display_record.php?ID = CRD42020168314). In addition, the review protocol has been published (Kwasnicka et al., 2021).

### Inclusion criteria

This review focused on the following five key health behaviours in healthy adults (i.e., non-clinical populations) aged 18 + years:

1. physical activity and sedentary behaviour, including the interruption of sitting time;
2. dietary behaviour, including snacking and fruit and vegetable consumption;
3. alcohol consumption, including binge drinking;
4. tobacco smoking, including cigarette-, cigar- and pipe smoking;
5. Sexual health behaviour, including contraceptive and condom use.

To limit the review scope and as several available reviews have focused on EMA studies in specific clinical populations (e.g., borderline personality disorder, psychotic disorder, binge eating, bulimia nervosa, schizophrenia, chronic pain), we opted to include only non-clinical populations. Studies that recruited individuals with overweight or obesity were judged as non-clinical and therefore included, given that 39% of adults globally meet criteria for overweight or obesity, with most Western countries averaging above 50% (World Health Organisation, 2021). We included studies that involved individuals with a diagnosed mental or physical health condition, providing that they were not specifically recruited into the study based on a mental or physical health condition. We also included studies in which a behavioural or pharmacological intervention was delivered, providing that participants were asked to complete free-living EMAs.

To the authors’ knowledge, there is no consensus definition of EMAs; therefore, we opted for an inclusive approach and included studies with repeated (i.e., two or more) within-day, daily or weekly assessments of psychological or contextual predictors and behaviours. We reasoned that the frequency of the EMAs needed to plausibly match how the target behaviour (and psychological and contextual predictors) theoretically or empirically unfolds over time (e.g., daily assessments of steps, weekly assessments of gym class attendance if the class is undertaken only once a week). To be included, studies needed to assess the target behaviour and at least one psychological or contextual variable through EMAs, and to have reported at least one within- or between-person predictor-behaviour association. In this review, we defined psychological variables as emergent properties of a distributed network of neurons, including cognitions (e.g., beliefs, attitudes, goals), emotions (e.g., negative affect, cravings) and processes operating on these (e.g., self-regulation, learning), which are linked to behaviour (Fried, 2017). We defined contextual variables as any potential environmental (i.e., social or physical) influences on behaviour, including the presence of other people, weather, or the availability of unhealthy foods/tobacco/alcohol. The psychological and contextual variables were closely assessed by the reviewers as to their suitability for inclusion/exclusion in the review. Studies reporting associations between behaviours and psychological consequences (e.g., the association of physical activity and positive affect) were included providing that they also reported at least one predictor-behaviour association (e.g., the association of positive affect with physical activity). Studies were included if they used self-report or physiological measures of psychological or contextual predictors (e.g., cortisol or heart rate variability to capture stress) or behaviours (e.g., accelerometer data to capture physical activity). No restrictions on geographical location or publication date were applied.

### Exclusion criteria

Studies only focusing on purchasing behaviours were excluded if they did not include any other relevant behaviour. Studies not published in English or where no full text could be obtained were not included. Behaviour-behaviour associations (e.g., the relationship between physical activity and eating behaviour) were not considered in this review.

### Search methods for identification of studies

#### Electronic searches

We searched Ovid MEDLINE, Embase, PsycINFO and Web of Science (see the Supplementary Materials for the full search strategy). Terms were searched for in titles and abstracts as free text or index terms (e.g., Medical Subject Headings), as appropriate. We combined two groups of terms, the first with terms relevant to EMAs and within-person study designs; the second with terms relevant to the five key health behaviours. Electronic and hand searches were conducted in January 2020 and updated on February 28th, 2021. The search was restricted to human studies written in English that were published in peer-reviewed journals.

#### Searching for other sources

Reference lists of available systematic reviews of EMA studies were hand searched and expertise within the review team was used to identify additional articles of interest.

### Data collection and analysis

#### Selection of studies

**Figure 1.**
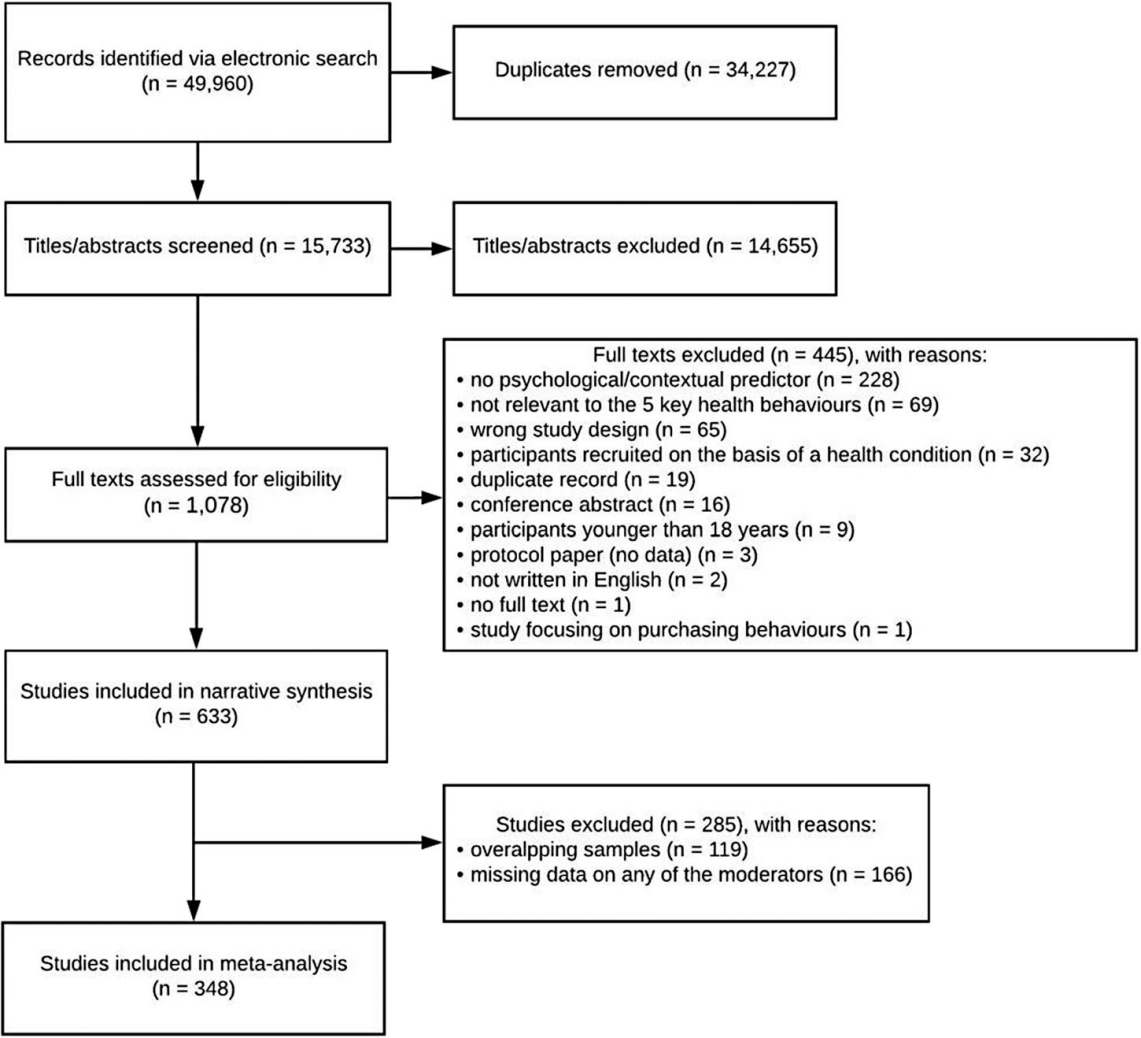
PRISMA flow chart.

Identified articles were merged using Covidence (www.covidence.org) and duplicate records were removed. Three reviewers (OP, JK, DKw) independently screened titles and abstracts (yes, maybe, no) against the pre-specified inclusion criteria. Authors were e-mailed to request access to full texts which could not be obtained electronically. Full texts were independently screened by two reviewers from the author team (yes, no). Discrepancies were resolved by three reviewers (OP, JK, DKw), consulting the other team members if needed. We did not calculate inter-rater reliability. In line with the PRISMA guideline, reasons for exclusion were recorded at the full text stage and are listed in Figure 1.

#### Data extraction and management

A data extraction form was developed in Microsoft Excel by the review leads in collaboration with the other team members. Data were extracted by one reviewer from the author team, with 20% of studies double checked for accuracy and completeness by a second reviewer from the author team. Discrepancies were resolved by the two reviewers involved in the data extraction and checking, consulting the other team members if needed. We did not calculate inter-rater reliability. Data were extracted on study description, health behaviour(s), participant characteristics, study design, EMA characteristics and adherence, and psychological and contextual predictors (see Kwasnicka et al., 2021, for further details). EMA adherence was defined as the average percentage EMAs completed (nominator) out of the available EMAs (denominator) across the study sample (Kwasnicka et al., 2021).

#### Quality appraisal

As a specific quality appraisal tool for EMA studies is currently not available, we devised a bespoke tool specifically for the purposes of this review based on previous literature (including the CREMAS checklist) (Liao et al., 2016; Stone & Shiffman, 2002). The quality appraisal tool was piloted by the review team and included the following four criteria: (1) rationale for the EMA design; (2) whether an a priori power analysis had been conducted; (3) adherence to the EMAs; and (4) treatment of missingness (see the Supplementary Materials, Table S1). In line with the Effective Public Health Practice Project quality assessment tool (Armijo-Olivo et al., 2012), we rated each of the four criteria as ‘Strong’, ‘Moderate’ or ‘Weak’. As each criterion refers to a different aspect of study quality, we did not produce an overall study quality rating for each study. The quality appraisal was performed by one reviewer from the author team, with 20% of studies double checked by a second reviewer from the author team. Discrepancies were resolved by the two reviewers involved in the data extraction and checking, consulting the other team members if needed. We did not calculate inter-rater reliability. Data synthesis.

A narrative (descriptive) synthesis was conducted to summarise the theoretical and methodological aspects of the EMA studies, first across all included studies and next split by target behaviour.

To aid interpretation and prior to summarising the psychological and contextual predictors assessed, we coded the identified constructs against the following higher-order categories, developed by three reviewers (OP, JK, DKw) based on the Theoretical Domains Framework (Atkins et al., 2017; Michie et al., 2005). The Theoretical Domains Framework (TDF) was developed through consensus methodology, with a view to integrating the many available behaviour change theories and theoretical constructs into a single framework, thus making theory more accessible to researchers and practitioners (Atkins et al., 2017; Michie et al., 2005). We used the following TDF-based, higher-order categories: ‘feeling states – unspecified’, ‘positive feeling states’, ‘negative feeling states’, ‘momentary trait manifestations and physical states’, ‘motivation and goals’, ‘beliefs about capabilities’, ‘beliefs about consequences’, ‘behavioural regulation’, ‘memory, attention and decision processes’, ‘social influences’, ‘environmental context and physical/environmental resources’ and ‘nature of the behaviour’ (see ‘Data statement’ for a link to the dictionary used). The psychological and contextual variables identified across the included studies were coded by one reviewer (OP) and double checked by two reviewers (DKw and JK). Discrepancies were resolved through discussion among three reviewers (OP, JK and DKw). Following an identical procedure, the identified funders were coded against the following higher-order categories: research/government, society, charity, university/health institution, industry or no funding reported (see ‘Data statement’ for a link to the dictionary used).

Second, although we did not systematically extract information on overlapping samples across included studies at the time of data extraction, we returned to the dataset to identify such samples using the following approach: (i) two reviewers (DP and FN) flagged studies with identical sample sizes and identical sample mean ages; and (ii) checked the author list for overlaps in co-authorship. Where (i) and (ii) were satisfied, studies were coded as having an overlapping sample. Where an overlap in co-authorship was not identified, the article full texts were further checked. Next, the ‘General Comments’ column in the data extraction sheet (used by reviewers to highlight any queries) was screened for any mention of overlapping samples, and where this was the case, this was confirmed by checking if the samples in the articles were the same or a subsample of each other. Finally, where the first approach brought up sample sizes and mean ages that were very close but not identical, the articles were further screened to check for overlapping samples. Studies with overlapping samples were excluded prior to the meta-analysis, keeping the earliest record of a study using each sample.

We then conducted a series of uni- and multivariable random-effects meta-analyses to estimate the pooled percentage adherence across included studies and to examine whether adherence varies depending on study setting, study population, whether an incentive(s) was provided, target behaviour, EMA delivery mode, EMA sampling frequency, EMA sampling method, whether an adherence cut-off was applied, year of publication, or study duration (in days), with some moderator levels collapsed due to low cell counts (see the Supplementary Materials, Table S2, for the moderator coding). Studies with missing data on any of the moderator variables were excluded from the uni- and multivariable meta-analyses. We did not have pre-specified hypotheses regarding potential moderators of EMA adherence; all variables were entered simultaneously into a multivariable random-effects model. Analyses were conducted in RStudio using the *metafor* package and with the estimator set to restricted maximum-likelihood (Viechtbauer, 2010). To aid interpretation, we did not apply any transformations of the raw percentages prior to meta-analysis. The *I^2^* statistic was used to quantify the between-study heterogeneity but we did not deem it useful to assess the potential for publication bias via, for example, Egger’s test given EMA researchers often apply adherence cut-offs for inclusion (which was already captured descriptively). Due to the large number of included studies, forest plots for each target behaviour were produced.

We had specified in the pre-registered study protocol that we aimed to synthesise predictor-behaviour associations using random effects meta-analyses, grouped by target behaviour (Kwasnicka et al., 2021). However, due to the length of the present review and the desire to describe predictor-behaviour associations in more depth, we opted instead to present such results as part of smaller, behaviour-specific sub-reviews (e.g., https://osf.io/49uqf/; https://osf.io/p2b65/), which are currently in progress.

## Results

After removing duplicates, 15,733 records were identified, with 1,078 studies carried forward to the full text screening. A total of 633 studies were included in the narrative synthesis, with 348 studies included in the meta-analysis to examine moderators of EMA adherence (see Figure 1).

### Study characteristics

Table 1 summarises the study characteristics of the included studies. Most studies focused on physical activity (187/633; 29.5%), followed by alcohol (175/633; 27.6%), smoking (139/633; 22.0%), dietary behaviour (111/633; 17.5%) and sexual health behaviour (21/633; 3.3%). Most studies were conducted in the United States (441/633; 70.1%), followed by Germany (32/633; 5.1%) and Australia (31/633; 4.9%; see Table 1 and Supplemental Materials, Figure S1). With the exception of the studies focused on sexual health behaviour, there appeared to be an increasing trend in the number of studies published over time (see Supplementary Materials, Figure S2).

**Table 1.** Characteristics of included EMA studies.

	Overall (*N *= 633)	Physical activity (*N *= 187, 29.5%)	Alcohol (*N *= 175,27.6%)	Smoking (*N *= 139,22.0%)	Healthy eating (*N *= 111,17.5%)	Sexual health (*N *= 21, 3.3%)
**Country**						
- United States	441 (69.7%)	103 (55.1%)	148 (84.6%)	112 (80.6%)	57 (51.4%)	21 (100.0%)
- Germany	32 (5.1%)	21 (11.2%)	1 (0.6%)	2 (1.4%)	8 (7.2%)	0 (0.0%)
- Australia	31 (4.9%)	7 (3.7%)	2 (1.1%)	4 (2.9%)	18 (16.2%)	0 (0.0%)
- United Kingdom	25 (3.9%)	13 (7.0%)	4 (2.3%)	2 (1.4%)	6 (5.4%)	0 (0.0%)
- Switzerland	24 (3.8%)	11 (5.9%)	2 (1.1%)	11 (7.9%)	0 (0.0%)	0 (0.0%)
- Canada	20 (3.2%)	10 (5.3%)	5 (2.9%)	0 (0.0%)	5 (4.5%)	0 (0.0%)
- Netherlands	18 (2.8%)	2 (1.1%)	7 (4.0%)	3 (2.2%)	6 (5.4%)	0 (0.0%)
- Austria	5 (0.8%)	0 (0.0%)	0 (0.0%)	0 (0.0%)	5 (4.5%)	0 (0.0%)
- China	5 (0.8%)	3 (1.6%)	1 (0.6%)	0 (0.0%)	1 (0.9%)	0 (0.0%)
- Not reported	11 (1.7%)	6 (3.2%)	3 (1.7%)	2 (1.4%)	0 (0.0%)	0 (0.0%)
- Korea, South	3 (0.5%)	2 (1.1%)	0 (0.0%)	1 (0.7%)	0 (0.0%)	0 (0.0%)
- Japan	2 (0.3%)	1 (0.5%)	0 (0.0%)	0 (0.0%)	1 (0.9%)	0 (0.0%)
- Multiple	2 (0.3%)	2 (1.1%)	0 (0.0%)	0 (0.0%)	0 (0.0%)	0 (0.0%)
- New Zealand	2 (0.3%)	0 (0.0%)	1 (0.6%)	0 (0.0%)	1 (0.9%)	0 (0.0%)
- Poland	2 (0.3%)	1 (0.5%)	0 (0.0%)	0 (0.0%)	1 (0.9%)	0 (0.0%)
- Belgium	1 (0.2%)	1 (0.5%)	0 (0.0%)	0 (0.0%)	0 (0.0%)	0 (0.0%)
- Brazil	1 (0.2%)	1 (0.5%)	0 (0.0%)	0 (0.0%)	0 (0.0%)	0 (0.0%)
- Cote d’Ivoire	1 (0.2%)	1 (0.5%)	0 (0.0%)	0 (0.0%)	0 (0.0%)	0 (0.0%)
- Estonia	1 (0.2%)	0 (0.0%)	0 (0.0%)	0 (0.0%)	1 (0.9%)	0 (0.0%)
- Finland	1 (0.2%)	1 (0.5%)	0 (0.0%)	0 (0.0%)	0 (0.0%)	0 (0.0%)
- France	1 (0.2%)	0 (0.0%)	1 (0.6%)	0 (0.0%)	0 (0.0%)	0 (0.0%)
- India	1 (0.2%)	0 (0.0%)	0 (0.0%)	1 (0.7%)	0 (0.0%)	0 (0.0%)
- Serbia	1 (0.2%)	0 (0.0%)	0 (0.0%)	1 (0.7%)	0 (0.0%)	0 (0.0%)
- Sweden	1 (0.2%)	1 (0.5%)	0 (0.0%)	0 (0.0%)	0 (0.0%)	0 (0.0%)
- Taiwan	1 (0.2%)	0 (0.0%)	0 (0.0%)	0 (0.0%)	1 (0.9%)	0 (0.0%)
**Research/government funding**						
- Yes	407 (64.3%)	93 (49.7%)	142 (81.1%)	106 (76.3%)	52 (46.8%)	14 (66.7%)
- No	226 (35.7%)	94 (50.3%)	33 (18.9%)	33 (23.7%)	59 (53.2%)	7 (33.3%)
**Society funding**						
- No	594 (93.8%)	170 (90.9%)	171 (97.7%)	128 (92.1%)	104 (93.7%)	21 (100.0%)
- Yes	39 (6.2%)	17 (9.1%)	4 (2.3%)	11 (7.9%)	7 (6.3%)	0 (0.0%)
**Charity funding**						
- No	597 (94.3%)	180 (96.3%)	168 (96.0%)	121 (87.1%)	108 (97.3%)	20 (95.2%)
- Yes	36 (5.7%)	7 (3.7%)	7 (4.0%)	18 (12.9%)	3 (2.7%)	1 (4.8%)
**University/health institution funding**						
- No	533 (84.2%)	156 (83.4%)	149 (85.1%)	127 (91.4%)	85 (76.6%)	16 (76.2%)
- Yes	100 (15.8%)	31 (16.6%)	26 (14.9%)	12 (8.6%)	26 (23.4%)	5 (23.8%)
**Industry funding**						
- No	611 (96.5%)	178 (95.2%)	173 (98.9%)	132 (95.0%)	107 (96.4%)	21 (100.0%)
- Yes	22 (3.5%)	9 (4.8%)	2 (1.1%)	7 (5.0%)	4 (3.6%)	0 (0.0%)
**No funding**						
- No	495 (78.2%)	119 (63.6%)	165 (94.3%)	119 (85.6%)	76 (68.5%)	16 (76.2%)
- Yes	138 (21.8%)	68 (36.4%)	10 (5.7%)	20 (14.4%)	35 (31.5%)	5 (23.8%)
**Study design**						
- Observational	533 (84.2%)	162 (86.6%)	166 (94.9%)	87 (62.6%)	97 (87.4%)	21 (100.0%)
- Interventional	100 (15.8%)	25 (13.4%)	9 (5.1%)	52 (37.4%)	14 (12.6%)	0 (0.0%)
**Intervention level**						
- Not applicable	533 (84.2%)	162 (86.6%)	166 (94.9%)	87 (62.6%)	97 (87.4%)	21 (100.0%)
- Between-person (group-level)	81 (12.8%)	17 (9.1%)	8 (4.6%)	45 (32.4%)	11 (9.9%)	0 (0.0%)
- Within-person (individual-level)	15 (2.4%)	7 (3.7%)	1 (0.6%)	5 (3.6%)	2 (1.8%)	0 (0.0%)
- Mixed	4 (0.6%)	1 (0.5%)	0 (0.0%)	2 (1.4%)	1 (0.9%)	0 (0.0%)
**Population type**						
- General population	272 (43.0%)	56 (29.9%)	63 (36.0%)	105 (75.5%)	44 (39.6%)	4 (19.0%)
- Students	197 (31.1%)	56 (29.9%)	93 (53.1%)	7 (5.0%)	36 (32.4%)	5 (23.8%)
- Other	74 (11.7%)	32 (17.1%)	13 (7.4%)	23 (16.5%)	5 (4.5%)	1 (4.8%)
- Older adults	27 (4.3%)	25 (13.4%)	2 (1.1%)	0 (0.0%)	0 (0.0%)	0 (0.0%)
- Overweight/obese	25 (3.9%)	7 (3.7%)	0 (0.0%)	0 (0.0%)	18 (16.2%)	0 (0.0%)
- Heterosexual couples	13 (2.1%)	4 (2.1%)	3 (1.7%)	4 (2.9%)	2 (1.8%)	0 (0.0%)
- Men who have sex with men	11 (1.7%)	0 (0.0%)	0 (0.0%)	0 (0.0%)	0 (0.0%)	11 (52.4%)
- Not reported	9 (1.4%)	2 (1.1%)	1 (0.6%)	0 (0.0%)	6 (5.4%)	0 (0.0%)
- Physically inactive	5 (0.8%)	5 (2.7%)	0 (0.0%)	0 (0.0%)	0 (0.0%)	0 (0.0%)
**Sample size**						
- Not reported	32 (5.1%)	16 (8.6%)	2 (1.1%)	1 (0.7%)	11 (9.9%)	2 (9.5%)
- Median	100.0	93.0	132.0	104.5	77.0	116.0
- IQR	62.0, 200.0	61.5, 146.0	83.0, 296.0	61.2, 248.0	50.0, 146.2	100.0, 203.0
**Age**						
- Not reported	39 (6.2%)	19 (10.2%)	9 (5.1%)	6 (4.3%)	4 (3.6%)	1 (4.8%)
- Median	31.1	34.3	20.9	40.5	30.0	27.1
- IQR	21.0, 41.2	23.3, 46.2	19.3, 26.9	33.9, 43.2	21.1, 40.2	24.8, 28.1
**% Female**						
- Not reported	21 (3.3%)	5 (2.7%)	1 (0.6%)	11 (7.9%)	4 (3.6%)	0 (0.0%)
- Median	58.0	66.0	54.0	51.0	83.0	0.0
- IQR	50.0, 73.5	55.8, 75.0	50.0, 60.9	45.0, 58.0	68.8, 100.0	0.0, 49.5
**% White ethnicity**						
- Not reported	196 (31.0%)	89 (47.6%)	20 (11.4%)	39 (28.1%)	46 (41.4%)	2 (9.5%)
- Median	76.5	73.8	82.0	72.0	70.9	82.0
- IQR	57.0, 87.0	49.3, 87.0	69.5, 89.0	37.8, 87.0	50.0, 78.6	70.5, 84.4
**% University education**						
- Not reported	419 (66.1%)	88 (47.1%)	147 (84.0%)	97 (69.8%)	75 (67.6%)	12 (57.1%)
- Median	42.3	0.0	53.0	44.8	43.9	51.3
- IQR	0.0, 69.7	0.0, 67.0	30.1, 70.7	34.0, 74.3	38.9, 60.2	44.0, 60.0
**Incentive schedule**						
- Not reported	192 (30.3%)	65 (34.8%)	46 (26.3%)	47 (33.8%)	31 (27.9%)	3 (14.3%)
- Multiple	130 (20.5%)	26 (13.9%)	56 (32.0%)	14 (10.1%)	26 (23.4%)	8 (38.1%)
- Flat payment based on study completion	98 (15.5%)	16 (8.6%)	21 (12.0%)	35 (25.2%)	25 (22.5%)	1 (4.8%)
- Other	65 (10.3%)	25 (13.4%)	8 (4.6%)	21 (15.1%)	11 (9.9%)	0 (0.0%)
- Payment per EMA	56 (8.8%)	9 (4.8%)	23 (13.1%)	18 (12.9%)	0 (0.0%)	6 (28.6%)
- Course credit	34 (5.4%)	16 (8.6%)	8 (4.6%)	1 (0.7%)	8 (7.2%)	1 (4.8%)
- Flat payment irrespective of study completion	29 (4.6%)	11 (5.9%)	8 (4.6%)	3 (2.2%)	7 (6.3%)	0 (0.0%)
- None	18 (2.8%)	15 (8.0%)	1 (0.6%)	0 (0.0%)	1 (0.9%)	1 (4.8%)
- Prize draw	11 (1.7%)	4 (2.1%)	4 (2.3%)	0 (0.0%)	2 (1.8%)	1 (4.8%)
						

Studies primarily received funding from research/government organisations (407/633; 64.3%). Just over one fifth of studies did not report any specific funding received (138/633; 21.8%).

Within the alcohol studies, most focused on the number of drinks (118/175; 67.4%), followed by drinking events (34/175; 19.4%), binge drinking events (11/175; 6.3%), or units of alcohol (8/175; 4.6%). Within the physical activity studies, most focused on moderate-to-vigorous intensity physical activity (MVPA; continuous or binary; 58/187; 31.0%), followed by more generic activities with at least light intensity (continuous or binary; 30/187; 16.0%), metabolic units (METs; 20/187; 10.7%), walking or step counts (17/187; 9.1%), sedentary behaviour (15/187; 8.0%), energy expenditure (13/187; 7.0%), active leisure (12/187; 6.4%), or physical activity counts per minute (11/187; 5.9%). Within the smoking studies, most focused on lapses (binary) (57/139; 41.0%), or cigarettes smoked (55/139; 39.6%). Within the dietary behaviour studies, most focused on snacking (28/111; 25.2%), followed by general food intake (23/111; 20.7%), binge eating (binary) (18/111; 16.2%), dieting (11/111; 9.9%), dietary lapse (binary) (10/111; 9.0%), fruit and vegetable consumption (10/111; 9.0%), or sugar and fat consumption (4/111; 3.6%). Within the sexual health behaviour studies, most focused on condom use (18/21; 85.7%).

Studies reported a median (Q1, Q3) sample size of 100.0 (62.0, 200.0) and included participants aged a median of 31.1 (21.0, 41.2) years. Studies included a median of 58.0% (50.0%, 73.5%) women, with 76.5% (57.0%, 87.0%) of participants identifying as White ethnicity, and 42.3% (0.0%, 69.7%) with a university degree. Most studies recruited participants from the general population (272/633; 43.0%) or student samples (197/633; 31.2%). Most studies used observational designs (533/633; 84.2%), with the remaining studies using interventional designs (100/633; 15.8%). Of the studies using interventional designs, most deployed between-person designs (i.e., an intervention was tested between two or more groups) (81/100; 81.0%), followed by within-person designs (i.e., an intervention was tested between different days/momentary states within the same individual) (15/100; 15.0%) or ‘mixed’ designs (i.e., a combination of between- and within-person designs) (4/100; 4.0%). Most studies reported some form of incentive for participation or data completion, including multiple incentives (129/633; 20.4%), flat payment based on study completion (98/633; 15.5%), payment per EMA (56/633; 8.9%), course credit (34/633; 5.4%), flat payment irrespective of study completion (30/633; 4.8%), or entering participants into a prize draw (11/633; 1.7%), whereas 30.3% (192/633) did not report whether or not there were any incentives for participation or data completion.

### EMA characteristics

Characteristics of the EMA study designs are summarised in Table 2. The median (Q1, Q3) study duration was 14.0 (7.0, 30.0) days, with a range of 1–738 days. A minority of studies (63/633; 10.0%) deployed a measurement burst design, with a median (Q1, Q3) of 3.0 (2.0, 4.5) bursts. Most studies provided all participants with a study specific EMA device (313/633; 50.2%). EMAs were primarily delivered via handheld devices (139/633; 22.0%), website/online (132/633; 20.9%) or mobile phone/smartphone apps (132/633; 20.9%). The most commonly used EMA sampling frequency was daily (238/633; 38.4%). The most commonly used EMA sampling method was ‘multiple’ (e.g., a combination of at least two sampling methods) (259/633; 41.1%), followed by fixed sampling (e.g., every evening) (200/633; 31.7%), signal contingent (random) sampling (74/633; 11.7%), signal contingent (fixed) sampling (57/633; 9.0%) and event contingent sampling (36/633; 5.7%). The median (Q1, Q3) percentage of EMA adherence was 83.7% (76.3%, 90.8%). A substantial minority of studies (239/633; 38.1%) reported using an adherence cut-off for inclusion of participants in the data analyses.

**Table 2.** EMA characteristics.

	Overall (N = 633)	Physical activity (*N *= 187)	Alcohol (*N *= 175)	Smoking (*N *= 139)	Healthy eating (*N *= 111)	Sexual health (*N *= 21)
**Study duration (days)**						
- Not reported	7 (1.1%)	1 (0.5%)	3 (1.7%)	3 (2.2%)	0 (0.0%)	0 (0.0%)
- Median	14.0	14.0	21.0	21.0	10.0	30.0
- IQR	7.0, 30.0	7.0, 28.0	14.0, 30.5	14.0, 30.0	7.0, 14.0	30.0, 42.0
**Burst design**						
- No	570 (90.0%)	166 (88.8%)	148 (84.6%)	135 (97.1%)	103 (92.8%)	18 (85.7%)
- Yes	63 (10.0%)	21 (11.2%)	27 (15.4%)	4 (2.9%)	8 (7.2%)	3 (14.3%)
**Number of bursts**						
- Not Applicable	570 (90.0%)	166 (88.8%)	148 (84.6%)	135 (97.1%)	103 (92.8%)	18 (85.7%)
- Median	3.0	3.0	4.0	2.5	3.0	2.0
- IQR	2.0, 4.5	3.0, 3.0	3.0, 6.0	2.0, 3.2	2.0, 3.5	2.0, 4.0
**% Own device**						
- None	313 (49.4%)	94 (50.2%)	59 (33.7%)	100 (71.9%)	59 (53.2%)	1 (4.8%)
- All participants	217 (34.3%)	45 (24.1%)	97 (55.4%)	27 (19.4%)	35 (31.5%)	13 (61.9%)
- Not applicable	46 (7.3%)	15 (8.0%)	13 (7.4%)	6 (4.3%)	11 (9.9%)	1 (4.8%)
- Some participants	30 (4.7%)	21 (11.2%)	3 (1.7%)	0 (0.0%)	3 (2.7%)	3 (14.3%)
- Not reported	21 (3.3%)	9 (4.8%)	3 (1.7%)	3 (2.2%)	3 (2.7%)	3 (14.3%)
- Majority of participants	6 (0.9%)	3 (1.6%)	0 (0.0%)	3 (2.2%)	0 (0.0%)	0 (0.0%)
**% EMA delivery mode**						
- Handheld device	139 (22.0%)	9 (4.8%)	43 (24.6%)	71 (51.1%)	15 (13.5%)	1 (4.8%)
- Website/online	132 (20.9%)	35 (18.7%)	69 (39.4%)	3 (2.2%)	14 (12.6%)	11 (52.4%)
- Mobile phone - app	132 (20.9%)	33 (17.6%)	21 (12.0%)	30 (21.6%)	42 (37.8%)	6 (28.6%)
- Multiple	104 (16.4%)	80 (42.8%)	10 (5.7%)	4 (2.9%)	9 (8.1%)	1 (4.8%)
- Pen-and-paper	36 (5.7%)	8 (4.3%)	8 (4.6%)	6 (4.3%)	13 (11.7%)	1 (4.8%)
- Not reported	26 (4.1%)	9 (4.8%)	4 (2.3%)	5 (3.6%)	8 (7.2%)	0 (0.0%)
- Mobile phone - SMS	22 (3.5%)	1 (0.5%)	7 (4.0%)	10 (7.2%)	3 (2.7%)	1 (4.8%)
- Mobile phone - multiple/other	19 (3.0%)	2 (1.1%)	6 (3.4%)	6 (4.3%)	5 (4.5%)	0 (0.0%)
- Other	16 (2.5%)	5 (2.7%)	6 (3.4%)	4 (2.9%)	1 (0.9%)	0 (0.0%)
- Wrist-worn device	6 (0.9%)	5 (2.7%)	0 (0.0%)	0 (0.0%)	1 (0.9%)	0 (0.0%)
- Hip/thigh-worn device	1 (0.2%)	0 (0.0%)	1 (0.6%)	0 (0.0%)	0 (0.0%)	0 (0.0%)
**% EMA frequency**						
- Other (e.g., event contingent)	14 (2.2%)	5 (2.7%)	2 (1.1%)	0 (0.0%)	5 (4.5%)	4 (19.0%)
- Hourly	12 (1.9%)	6 (3.2%)	2 (1.1%)	2 (1.4%)	2 (1.8%)	0 (0.0%)
- Multiple times/day	349 (55.1%)	80 (42.8%)	63 (36.0%)	119 (85.6%)	82 (73.9%)	3 (14.3%)
- Daily	238 (37.6%)	84 (44.9%)	101 (57.7%)	18 (12.9%)	21 (18.9%)	14 (66.7%)
- Weekly	20 (3.2%)	12 (6.4%)	7 (4.0%)	0 (0.0%)	1 (0.9%)	0 (0.0%)
**% Adherence**						
- Not reported	157 (24.8%)	34 (18.2%)	40 (22.9%)	48 (34.5%)	32 (28.8%)	3 (14.3%)
- Median	83.7	84.6	84.0	81.3	84.5	79.8
- IQR	76.3, 90.8	77.0, 92.0	76.9 - 90.8	75.8, 88.0	78.0, 92.0	78.9, 86.9
**Adherence cut-off**						
- No	376 (59.4%)	111 (59.3%)	112 (64.7%)	68 (48.9%)	68 (61.3%)	17 (81.0%)
- Yes	239 (37.8%)	63 (33.7%)	61 (35.3%)	68 (48.9%)	43 (38.7%)	4 (19.0%)
- Not reported	18 (2.8%)	13 (7.0%)	2 (1.1%)	3 (2.2%)	0 (0.0%)	0 (0.0%)
**% EMA sampling method**						
Event contingent	36 (5.7%)	6 (3.2%)	7 (4.0%)	7 (5.0%)	16 (14.4%)	0 (0.0%)
Fixed (e.g., every evening)	200 (31.6%)	51 (27.3%)	90 (51.4%)	17 (12.2%)	26 (23.4%)	16 (76.2%)
Multiple	259 (40.9%)	101 (54.0%)	44 (25.1%)	82 (59.0%)	30 (27.0%)	2 (9.5%)
Signal contingent-fixed timing	57 (9.0%)	10 (5.3%)	24 (13.7%)	5 (3.6%)	16 (14.4%)	2 (9.5%)
Signal contingent-random timing	74 (11.7%)	15 (8.0%)	9 (5.1%)	27 (19.4%)	23 (20.7%)	0 (0.0%)
Not reported	7 (1.1%)	4 (2.1%)	1 (0.6%)	1 (0.7%)	0 (0.0%)	1 (4.8%)

### Psychological and contextual predictors of the five key health behaviours

**Figure 2.**
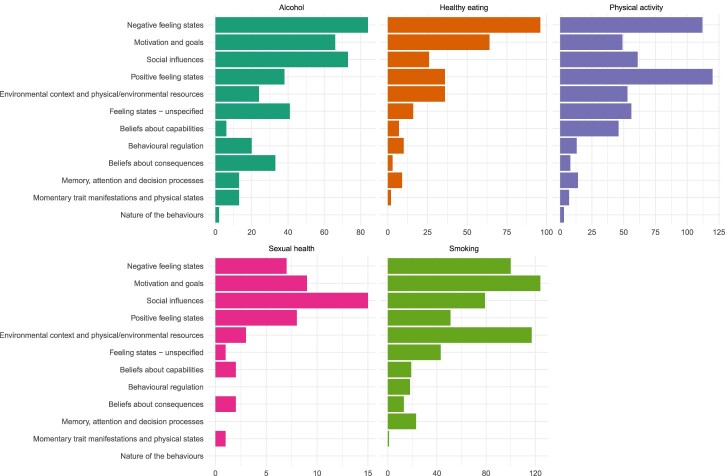
Frequency of psychological and contextual variables assessed using EMA methods, stratified by target behaviour.

Studies assessed a median (Q1, Q3) of 3 (2, 4) psychological or contextual predictors (range = 1-12). A total of 1,896 psychological and contextual predictors were recorded across the included studies. The most frequently assessed variables were ‘negative feeling states’ (399/1896; 21.0%) and ‘motivation and goals’ (312/1896; 16.5%), but this varied by target behaviour (see Figure 2). Within the physical activity studies, the most frequently examined constructs were ‘positive feeling states’ (120/542; 22.1%) and ‘negative feeling states’ (112/542; 20.7%). Within the alcohol studies, the most frequently examined constructs were ‘negative feeling states’ (84/413; 20.3%) and ‘social influences’ (73/413; 17.7%). Within the dietary behaviour studies, the most frequently examined constructs were ‘negative feeling states’ (96/305; 31.5%) and ‘motivation and goals’ (64/305; 21.0%). The sexual health behaviour studies focused primarily on ‘social influences’ (15/48; 31.3%) and ‘motivation and goals’ (9/48; 18.8%), whereas the smoking studies focused on ‘motivation and goals’ (124/588; 21.1%) and ‘environmental context and physical/environmental resources’ (117/588; 19.9%).

Overall, of the psychological and contextual predictors assessed across the included studies, a substantial minority (789/1896; 41.6%) were measured with multiple items (vs. a single item or not reported), with 297 studies (297/633; 46.9%) using solely multiple item-scales to capture psychological or contextual predictors of interest. Of the psychological and contextual predictors assessed across the included studies, just over a third (633/1896; 33.4%) were reported to have been measured with items for which there was a ‘precedent’ (i.e., items that had previously been used in one or more EMA studies vs. items being developed specifically for the study or the item origin not being reported).

### EMA adherence

After removing studies with duplicate samples and those with missing data on any of the moderator variables of interest, a random-effects meta-analysis (*k* = 348) showed that the pooled percentage adherence was 81.4% (95% CI = 80.0%, 82.8%). However, there was substantial between-study heterogeneity (I^2 ^= 97.1%). The pooled percentage adherence did not vary markedly by target behaviour: physical activity (82.0%, 95% CI = 80.0%, 85.0%), alcohol (82.0%, 95% CI = 80.0%, 85.0%), dietary behaviour (82.0%, 95% CI = 78.0%, 85.0%), smoking (78.0%, 95% CI = 75.0%, 81.0%), and sexual health behaviour (79.0%, 95% CI = 73.0%, 84.0%). Forest plots for random-effects meta-analyses stratified by target behaviour are presented in the Supplementary Materials, Figures S3-S7.

In a subsequent, multivariable random-effects meta-analysis with moderators entered (*k* = 348), study population, EMA delivery mode, EMA sampling method, EMA device ownership and year of publication were significant moderators of EMA adherence (see Table 3). Specifically, greater adherence was observed in studies with student (vs. general) population samples, mobile phone/smartphone (vs. handheld device) EMA delivery, and event contingent (vs. fixed) EMA sampling. Reduced adherence was observed in studies with all/majority (vs. none) of participants using their own device and random (vs. fixed) EMA sampling. Since the first EMA publication included in the meta-analysis in 1987, for every decade until 2021, adherence decreased by 3.1%.

**Table 3.** Results from the multivariable random-effects adherence meta-analysis.

	Estimate*	Lower CI	Upper CI	*p*-value
intercept	0.757	0.619	0.895	**<0**.**001**
***Country (reference: Other)***				
US	0.024	−0.006	0.054	0.110
***Population (ref: General population)***				
Other	0.021	−0.014	0.056	0.241
Students	0.050	0.017	0.084	**0**.**003**
***Incentive schedule (ref: No incentive)***				
Incentive	0.054	−0.016	0.124	0.132
*** ***Not reported	0.033	−0.041	0.106	0.382
***Study design (ref: Observational)***				
Interventional	−0.023	−0.063	0.017	0.258
***EMA delivery mode (ref: Handheld device)***				
Mobile phone/smartphone	0.049	0.002	0.096	**0**.**042**
Multiple	0.042	−0.012	0.096	0.124
Not reported	0.075	−0.014	0.163	0.099
Other	−0.065	−0.159	0.030	0.179
Pen-and-paper	0.007	−0.076	0.089	0.877
Website/online	−0.019	−0.080	0.042	0.541
Wrist/hip/thigh-worn device	0.071	−0.058	0.20	0.281
***EMA sampling frequency (ref: Weekly)***				
Daily	0.062	−0.004	0.129	0.067
Hourly/multiple times per day	−0.007	−0.078	0.065	0.858
***EMA sampling method (ref: Fixed (e.g., every evening))***				
Event contingent	0.100	0.025	0.176	**0**.**009**
Multiple	0.019	−0.032	0.071	0.460
Not reported	−0.132	−0.318	0.055	0.167
Signal contingent – fixed timing	−0.013	−0.070	0.044	0.648
Signal contingent – random timing	−0.061	−0.118	−0.003	**0**.**038**
***Own device (ref: None)***				
All/majority of participants	−0.053	−0.091	−0.016	**0**.**005**
Some participants	−0.024	−0.085	0.037	0.436
Not applicable	0.018	−0.033	0.069	0.498
***Target behaviour (ref: Sexual health)***				
Alcohol	0.045	−0.025	0.115	0.204
Dietary behaviour	0.050	−0.029	0.128	0.215
Physical activity	0.037	−0.037	0.111	0.330
Smoking	0.037	−0.040	0.114	0.347
***Adherence cut-off (ref: No)***				
Not reported	0.004	−0.077	0.084	0.930
Yes	−0.008	−0.034	0.019	0.575
Year of publication**	−0.031	−0.060	−0.002	**0**.**037**
***Study duration in days (mean centered)***	0.000	−0.001	0.000	0.087

Note*.* Bolded values are significant at *p* < 0.05. CI = Confidence Interval. * Raw proportions. ** To aid interpretation, year of publication was centered (0 = 1987) and divided by 10 prior to analysis.

### Quality appraisal

The quality ratings are summarised in Table 4. Overall, studies generally received a ‘Strong’ rating (554/633; 87.7%) for Quality 1 (i.e., rationale for the EMA design provided), a ‘Weak’ rating (591/633; 93.4%) for Quality 2 (i.e., no *a priori* power analysis had been conducted), a ‘Strong’ rating (315/633; 49.8%) for Quality 3 (i.e., an average adherence rate of at least 80% to the EMA protocol) and a ‘Weak’ rating (455/633; 72.1%) for Quality 4 (i.e., no analysis of EMA missingness or controlling for potential missing mechanisms).

**Table 4.** Quality of included studies.

	Overall (N = 633)	Physical activity (*N *= 187)	Alcohol (*N *= 175)	Smoking (*N *= 139)	Healthy eating (*N *= 111)	Sexual health (*N *= 21)
**Quality 1 – Rationale for the EMA design**						
Weak	19 (3.0%)	7 (3.7%)	3 (1.7%)	3 (2.2%)	4 (3.6%)	2 (9.5%)
Moderate	60 (9.5%)	21 (11.3%)	12 (6.9%)	9 (6.5%)	14 (12.6%)	4 (19.0%)
Strong	554 (87.5%)	159 (85.0%)	160 (91.4%)	127 (91.4%)	93 (83.8%)	15 (71.4%)
**Quality 2 – Whether an a priori power analysis had been conducted**						
Weak	591 (93.4%)	164 (87.7%)	167 (95.4%)	133 (95.7%)	106 (95.5%)	21 (100.0%)
Moderate	6 (0.9%)	3 (1.6%)	2 (1.1%)	1 (0.7%)	0 (0.0%)	0 (0.0%)
Strong	36 (5.7%)	20 (10.7%)	6 (3.4%)	5 (3.6%)	5 (4.5%)	0 (0.0%)
**Quality 3 – Adherence to the EMAs**						
Weak	176 (27.8%)	40 (21.4%)	41 (23.4%)	58 (41.7%)	35 (31.5%)	2 (9.5%)
Moderate	142 (22.4%)	35 (18.7%)	45 (25.7%)	35 (25.2%)	18 (16.2%)	9 (42.9%)
Strong	315 (49.8%)	112 (59.9%)	89 (50.9%)	46 (33.1%)	58 (52.3%)	10 (47.6%)
**Quality 4 – Treatment of missingness**						
Weak	457 (72.2%)	126 (67.4%)	117 (66.9%)	110 (79.1%)	88 (79.3%)	16 (76.2%)
Moderate	97 (15.3%)	22 (11.8%)	43 (24.6%)	19 (13.7%)	11 (9.9%)	2 (9.5%)
Strong	79 (12.5%)	39 (20.8%)	15 (8.6%)	10 (7.2%)	12 (10.8%)	3 (14.3%)

## Discussion

This systematic review and meta-analysis summarises the state-of-the-art in EMA studies conducted in non-clinical populations and across five key health behaviours. We identified 633 studies that investigated psychological and/or contextual predictors of the health behaviours of interest, with most studies focused on physical activity or alcohol consumption. The number of EMA studies across all (except for sexual health) behaviours of interest appears to have increased over time; this likely reflects popularisation of the EMA method and elevated technological progress that facilitates real-time data collection (Gibbons, 2017).

### Study characteristics

Most of the included studies were conducted in the US, with a large proportion of participants having a university degree and identifying as White ethnicity. This aligns with research showing that much of our psychological science is based on what has been described as WEIRD populations (i.e., Western, Educated, Industrialised, Rich and Democratic) (Henrich et al., 2010). However, the included EMA studies reported a relatively equal gender split and most studies recruited participants from the general population rather than student cohorts (although the latter was also common).

Most included studies applied observational designs, suggesting that interventional designs are currently less common in EMA research. In addition, within the few identified interventional studies, most tested interventions in which allocation occurred between rather than within participants, suggesting that the latter design remains rare, as highlighted in reviews of N-of-1 studies (which typically harness EMAs) (Kwasnicka et al., 2019). Recently, researchers have demonstrated the potential of EMAs for first exploring participants’ behavioural patterns in context, followed by interventions tailored to the most important predictors identified in the observational phase (Kwasnicka et al., 2020).

EMAs were primarily delivered via technological tools, such as handheld devices or mobile phones/smartphones. More than half of studies provided all participants with a study specific EMA device, such as a handheld device or activity monitor. The most commonly used EMA sampling frequency was daily, and the most commonly used EMA sampling method was ‘multiple’ (e.g., a combination of at least two sampling methods such as event and signal contingent prompts).

### Psychological and contextual predictors of the five key health behaviours

The most frequently assessed psychological and contextual variables fit into the higher-order categories ‘negative feeling states’ and ‘motivation and goals’; however, this varied by target behaviour. For instance, the studies focused on sexual health behaviours primarily captured ‘social influences’ and ‘motivation and goals’. Our review also highlights that some construct domains from the Theoretical Domains Framework (Atkins et al., 2017) have been relatively understudied (e.g., ‘memory, attention, and decision processes’). Further planned behaviour-specific sub-reviews and meta-analyses will examine in depth the ways in which the identified constructs have been assessed for each health behaviour and pool data on predictor-behaviour associations to understand their relative importance (e.g., https://osf.io/49uqf/; https://osf.io/p2b65/). Our database of included EMA studies is openly available and we encourage other researchers to explore how different psychological and contextual variables have been assessed across the five health behaviours.

Just over 40% of psychological and contextual predictors were assessed with multiple (rather than single) items and just over a third were reported to have been measured with items for which there was a precedent. The Experience Sampling Methodology (ESM) Item Repository (https://www.esmitemrepositoryinfo.com/) and working group were established to progress EMA methodology and help researchers identify relevant EMA items. The repository includes a searchable database which allows researchers to identify if a given item has been used in a previous EMA study and future aims include psychometrically validating items in the repository.

### Moderators of EMA adherence

In the meta-analysis of moderators of EMA adherence, the pooled percentage adherence was high at around 80% and comparable across the five target behaviours. This is similar to numbers reported in previous reviews of EMA studies, which have ranged from 71.6% to 79.0% (Cain et al., 2009; Colombo et al., 2019; de Vries et al., 2021; Degroote et al., 2020; Heron et al., 2017; Jones et al., 2019; Schembre et al., 2018; Wen et al., 2017). However, substantial between-study heterogeneity was detected in our review.

Most studies reported providing some type of incentive for participation or data completion (e.g., flat payment based on study completion, payment per EMA, course credit). However, in the meta-analysis, there was no significant association between the receipt of an incentive and adhering better to the study protocol (vs. no incentive), which stands in contrast to other studies reporting that financial incentives in particular are associated with greater adherence (Giles et al., 2014). Possibly, adherence rate in EMA studies is not primarily related to extrinsic factors (e.g., financial incentives) as participants might be motivated due to intrinsic factors such as their interest in the real-life examination of their health behaviours. Similarly, studies that recruited students reported significantly greater EMA adherence, which may be related to students’ increased motivation to contribute to science (Jang, 2008).

Studies in which EMAs were delivered via mobile phones/smartphones reported significantly greater adherence than those using handheld devices, suggesting that phones are suitable for answering EMA prompts, as participants are used to carrying smartphones with them throughout the day (Statista, 2021). However, studies in which all or the majority of participants used their own device to respond to EMAs reported significantly lower adherence than when using a device provided by the research team. This may be interpreted to suggest that adding objects to participants’ environment (i.e., a dedicated study phone) – an unintended behaviour change technique (Michie et al., 2013) – may act as a method for increasing study adherence. It is also possible that other apps on participants’ own devices generated similar alerts, which may have interfered with their engagement with the EMA alerts.

We note that researchers also need to consider the environmental impact on buying new electronic devices for each EMA study (Chevance, Hekler, et al., 2020), which is often driven by incompatibilities between new EMA software and the operating systems in older smartphones. However, if we are aiming to achieve sustainability in EMA research, we need to take into consideration that data collection devices and the energy that they use to run are limited. We need to carefully weigh costs and benefits of using technology, including when to purchase new (as opposed to recycling old) devices. Reusing devices across studies and opting for energy saving devices/functionalities where possible (e.g., traditional short message service; SMS) (Dondyk et al., 2015) are some of the potential solutions for making EMA research more environmentally friendly.

In addition, year of publication was a significant moderator of adherence, such that the reported adherence to EMA schedules has reduced over time – on average by 3.1% per decade since 1987. It is possible that methodological advances have made it more straightforward to accurately detect adherence, with fewer opportunities to backfill EMAs when these are prompted by digital technologies (e.g., smartphones). As a further explanation, people’s digital environment (e.g., the frequency of notifications from multiple apps) has changed in recent years, potentially reducing attention to EMA prompts.

Study duration and sampling frequency were not significant moderators of EMA adherence. However, studies that used event contingent sampling reported significantly greater EMA adherence and those using random prompts reported significantly reduced adherence compared with fixed sampling (e.g., every evening). The former may simply be explained by participants reporting ‘in the moment’ (e.g., when smoking a cigarette) making it close to impossible to assess if the participant reported all occurrences of the behaviour; therefore, adherence rates are inflated in studies applying this type of sampling method. The latter may be explained by participants being unable to anticipate prompts, meaning they may be busy at times of randomly sent prompts.

### Quality appraisal

Most included studies did not provide an *a priori* power analysis to justify sample sizes at the within- or between-person level. This is similar to other psychology domains: for example, a recent review in the psychopathology domain found that only 2% of included studies reported a power calculation (Trull & Ebner-Priemer, 2020). Conducting sample size calculations for EMA studies is complex and requires various parameters to be estimated which can be difficult to know in advance without access to pilot data or previous studies that fully report model outputs. The latter is often absent, with random effects commonly omitted from papers and supplementary materials. Tutorials for how to conduct power analyses for EMA studies have been published (Bolger et al., 2012; Lafit et al., 2021); however, their use appears rather limited. In addition to the above issues relating to uncertainties about model parameters, off-the-shelf power analysis tools for EMA studies are not widely available in popular statistical software (but see, for example, Green & MacLeod, 2016 and Lafit et al., 2021 for available tools). Therefore, researchers often rely on ‘rules of thumb’ when making decisions about the sample size in EMA studies.

Most included studies did not interrogate reasons for EMA missingness or control for missing mechanisms in their analyses. Although some missing data are inevitable in EMA studies, the statistical techniques used to analyse clustered data require that data are missing at random or missing completely at random for these to be ‘ignorable’ within the analyses (Little & Rubin, 2019). Where data are missing not at random, both the process of interest and the process of missingness must be simultaneously modelled (Black et al., 2012). Researchers have, for example, used innovative methods such as unobtrusive ‘eavesdropping’ to understand factors associated with missed EMAs (Sun et al., 2020).

We strongly encourage EMA researchers to increase the methodological rigour and transparency of EMA research. We echo our colleagues’ call (Kirtley et al., 2021) for greater use of study pre-registrations, using a template for EMA research to register both prospective studies and secondary analyses of available data. In order to progress dynamic theory building and making the most out of EMA data, we also strongly encourage data sharing (e.g., via the Open Science Framework) and the sharing of questionnaire items (e.g., via the ESM Item Repository; https://www.esmitemrepositoryinfo.com/).

### Strengths

First, a key strength of this review is the comprehensive summary of the application of the EMA method since its inception and across five key health behaviours. Second, we provided an overview of psychological and contextual predictors examined across EMA studies, highlighting differences in focus across the five health behaviours and identifying gaps for future research. Third, we summarised moderators of EMA adherence. Fourth, there is currently no consensus on how to reliably determine the quality of EMA studies. We therefore opted to design a bespoke quality appraisal tool, drawing on available checklists. Although this was useful for the purposes of our review, the tool requires further optimisation prior to wider use. Other research teams are in the process of developing more comprehensive frameworks and quality assessment tools that can be used in future reviews of EMA studies (although these remain unpublished). Fifth, this review was conducted by an international team of researchers, with team members collaborating online throughout the research process. Sixth, we closely followed the principles of Open Science, including study pre-registration; publication of the review protocol; documentation of design and analytic decisions; and sharing the analytic code, procedures, and the underlying dataset for transparency and reuse (McKiernan et al., 2016). The authors strongly encourage other EMA researchers to use and update the electronic searches and the database of EMA studies.

### Limitations

First, some of the included studies are likely to have used overlapping samples. As we did not have resource to contact study authors, we attempted to identify articles using the same dataset by checking sample sizes and author names, and – where identified – removed studies with overlapping samples prior to conducting the meta-analysis. However, we may not have identified all such studies, thus potentially biasing the pooled estimates. The results should therefore be interpreted with caution.

Second, although our review provided an overview of theoretical and methodological aspects of EMA studies, we did not attempt to quantify potential reactivity effects (i.e., whether repeatedly responding to EMAs may lead to behaviour change) (Wilding et al., 2016). However, this has been explicitly studied in extant EMA reviews (König, Allmeta, et al., 2021).

Third, this review focused solely on non-clinical populations. We acknowledge that there is a large number of EMA studies conducted in clinical populations.

Fourth, we focused on five key health behaviours (due to their relationships with morbidity and mortality) and presented the results stratified by target behaviour. However, we acknowledge that some of the health behaviours of interest can usefully be split into further sub-behaviours (e.g., ‘movement behaviour’ tends to be split into physical activity and sedentary behaviour, ‘dietary behaviour’ tends to be split into several categories, including fruit and vegetable consumption, sugary beverage consumption, etc.), which are expected *a priori* to be differently associated with psychological and contextual variables. This will be further explored in a series of behaviour-specific sub-reviews, which will look at such questions in more depth.

Fifth, since initiating this project, a few similar reviews focusing on EMA adherence have been published (Ottenstein & Werner, 2021; Wrzus & Neubauer, 2022). The present review is unique in that it is the first to consider both the theoretical aspects of EMA studies (e.g., the psychological and contextual predictors assessed) and study quality across key health behaviours. Although many of the results presented here align with those in extant reviews (e.g., EMA adherence), and did not differ markedly by the target health behaviour, these remained empirical questions prior to the present review.

Sixth, and related to the above limitation, our updated search was conducted in February 2021 and many relevant EMA studies have likely been published since. The database of included studies, the search strategy and all relevant study materials are published open source and we strongly encourage other researchers to update the search and to make further use of the extracted data.

Seventh, due to the already wide scope of the current review, we did not search the grey literature (e.g., PhD theses, pre-prints, other unpublished sources). Additional relevant studies may therefore have been missed.

Finally, due to the cost of EMA data collection (e.g., participant burden, researcher time), researchers often collect data on many variables within a single study and subsequently use different variable sets for different papers. Therefore, it is plausible that the number of variables reported in the included studies did not correspond to the actual number of variables assessed. We strongly encourage EMA researchers to publish study protocols and fully anonymised datasets.’

### Wider implications and avenues for future research

Future EMA research would benefit from harnessing advancements in sensor technology to detect health behaviours and contexts/locations (e.g., using geo-location, ambient light, biomarkers such as cortisol or glucose) (Reichert et al., 2020) and applying novel methods such as micro-EMAs to reduce participant burden, increase EMA adherence and increase the precision of EMAs (Ponnada et al., 2021). In addition, we note that most EMA studies reviewed here relied on ‘rules of thumb’ to guide key study design decisions (e.g., study duration, assessment frequency).

Event and signal contingent designs serve different purposes in data collection. However, event contingent sampling is associated with greater EMA adherence due to limited opportunities to estimate the ‘true’ denominator (i.e., the actual event rate is unknown) and this leads to inflated adherence rates reported in studies using event contingent EMAs.

EMA studies allow researchers to test theories within individuals over time, and to build dynamic behaviour change theories. However, we note that few studies explicitly tested behaviour change theories or used EMAs to develop and validate dynamic theories (Hall & Fong, 2007). Drawing on recent developments in sensor technology, natural language processing, and pattern recognition (Naylor, 2018), we are now at an opportune time to design EMA studies that facilitate understanding of individuals in context and then devise interventions that enhance health behaviour change and maintenance.

Future EMA studies should consider, where appropriate, to move beyond observation and intervene at the within-person level, for example by deploying ‘just-in-time adaptive interventions’ (JITAIs). JITAIs can be defined as interventions providing the right type and amount of support, at the right time, by adapting intervention delivery to an individual’s changing psychological and contextual states (Nahum-Shani et al., 2016). Dynamic interventions such as JITAIs also have the potential to inform how psychological and contextual factors co-vary with health behaviours through their attempts at modification, and therefore present an exciting avenue for future research and theory development.

## Conclusions

This systematic review and meta-analysis of EMA studies conducted across five key health behaviours found that studies have largely focused on capturing negative feeling states and motivation and goals. Participants’ adherence to EMAs was high (around 80%) and did not differ by target behaviour but was higher in student (vs. general) samples, when EMAs were delivered via mobile phones (particularly when using a study provided phone), and when event contingent sampling was used (although this is due to artificially inflated adherence rates in such studies). The quality of future EMA studies could be improved by conducting a priori power analyses and better accounting for EMA missingness. Future work harnessing EMAs may benefit from moving from understanding and predicting behavioural patterns to designing dynamically tailored interventions and building dynamic health behaviour change theories.

## Review registration

The review protocol was registered with PROSPERO (CRD42020168314). Available from: www.crd.york.ac.uk/prospero/display_record.php?ID = CRD42020168314.

## Data Availability

The data underpinning the analyses are openly available via Zenodo: https://doi.org/10.5281/zenodo.5701127. The R code used for the analyses is openly available via GitHub: https://github.com/OlgaPerski/EMA_systematic_review
